# Learning Agility of Learning and Development Professionals in the Life Sciences Field During the COVID-19 Pandemic: Empirical Study

**DOI:** 10.2196/33360

**Published:** 2022-04-26

**Authors:** XinYun Peng, Nicole Wang-Trexler, William Magagna, Susan Land, Kyle Peck

**Affiliations:** 1 Department of Learning and Performance Systems Pennsylvania State University University Park, PA United States; 2 Vanguard Group, Inc Valley Forge, PA United States; 3 Siemens Healthineers Newark, DE United States

**Keywords:** COVID-19, learning agility, learning and development professionals, life sciences professionals, training and development, mixed methods

## Abstract

**Background:**

The COVID-19 pandemic has impacted the life sciences field worldwide. Life sciences organizations (eg, pharmaceutical and med-tech companies) faced a rapidly increasing need for vital medical products, patient support, and vaccine development. Learning and development (L&D) departments play a crucial role in life sciences organizations as they apply learning initiatives to organizational strategy within a constantly evolving sector. During the COVID-19 pandemic, the work of L&D professionals in life sciences organizations changed profoundly during the abrupt shift to remote work, since learning and training normally occur in a face-to-face environment. Given the complex and dynamic situation of the pandemic, both individuals and organizations needed to learn quickly and apply what they learned to solve new, unprecedented problems. This situation presents an opportunity to study how characteristics of learning agility were evidenced by life sciences organizations and individual employees in the remote working mode.

**Objective:**

In collaboration with Life Sciences Trainers & Educators Networks (LTEN), this study investigated the responses and learning agility of L&D professionals and their organizational leadership within the life sciences sector to the work changes due to the pandemic. The study answered the following questions: (1) How did L&D professionals in the life sciences sector respond to the changes in their work environment during the COVID-19 pandemic? (2) How did L&D professionals in the life sciences sector demonstrate learning agility during remote working?

**Methods:**

We adopted a mixed methods approach that included a semistructured interview and a survey. Participants who were life sciences or health care L&D practitioners and in relevant positions were recruited via email through the LTEN and its partner pharmaceutical, biotech, or medical devices organizations. Interviews with 12 L&D professionals were conducted between June and August 2020 through phone or online conferencing, covering 22 open-ended questions to stimulate ideas that could be explored further in the survey. The semistructured interview questions were grounded in theory on learning agility. In total, 4 themes were developed from the interviews, which formed the basis for developing the survey items. The subsequent survey regarding 4 specific themes was conducted from August to October 2020 using Qualtrics. Both interview and survey data were analyzed based on a learning agility framework.

**Results:**

Findings revealed generally positive organizational and individual responses toward the changes brought about by the pandemic. Results also indicated that a disruptive crisis, such as the shift from working in the office to working from home (WFH), required professionals’ learning agility to both self-initiate their own learning and to support the learning agility of others in the organization.

**Conclusions:**

This study was designed to better understand education and training in the life sciences field, particularly during the unique circumstances of the global COVID-19 pandemic. We put forward several directions for future research on the learning agility of L&D professionals in life sciences organizations.

## Introduction

During 2020 and 2021, almost every organization evolved and shifted to address the COVID-19 pandemic, including extensive numbers of employees working from home (WFH) due to lockdowns and shelter-in-place orders [1]. As the coronavirus continues to spread, many organizations (at the time of this writing) still have not set a date to return to their physical offices. Learning and development (L&D) professionals excel at understanding the organization’s future capability needs and identifying priorities and learning solutions for the organization. Therefore, they are playing a pivotal role in transitioning and implementing changes within their organizations. Recent L&D studies have investigated learning in the health and life sciences with informational technologies, virtual programs, or online platforms, as well as their feasibility and effectiveness during the pandemic [2-7]. Much of the recent pandemic-related research on health and life sciences is concerned with digital mental health, especially stress and depression trends [8-11]. However, there is little research on the learning agility of L&D professionals in the health and life sciences, particularly how they dealt with the abrupt change to remote working. We argue that the learning agility of L&D professionals is essential to the survival and growth of organizations. This study describes the learning agility of L&D professionals in the life sciences sector, as they encountered drastic changes in their job requirements due to COVID-19.

The concept of learning agility was coined by Lombardo and Eichinger [12]. It is defined as the willingness and ability to learn from experience and subsequently apply that learning to perform successfully under new or first-time conditions. Learning agility is tied closely to developmental job experiences and reflects the complexity of challenging jobs. As such, it is considered an early indicator of one’s potential and leadership effectiveness and therefore is used by organizations to identify and develop high-potential employees [13-15]. To elaborate on the Lombardo and Eichinger [12] definition, learning agility describes the following characteristics of a person: the willingness to adapt to new job requirements, the ability to continuously learn new things, to overcome difficulties, and to manage multiple, sometimes contradictory, tasks. Being mentally prepared for job requirements that are different and unfamiliar and being prepared to constantly learn new things for them are prerequisites of being agile. Effective leaders normally have a broad portfolio of leadership roles and can vary their performance of leadership skills, depending on the situation, showing high learning agility when encountering difficulties. In addition, existing and widely acknowledged theories of leadership tend to classify one’s leadership into contrasting categories, for example, task oriented versus relation oriented [16], directive versus participative [17], autocratic versus consultative [18], and transactional versus transformational [19]. Lombardo and Eichinger [12] proposed that effective leaders should be able to accommodate multiple opposing categories to react to dilemmas under multiple circumstances.

Learning agility is frequently raised in corporate conversations and business reports when discussing how the working mode for people changed so abruptly due to the COVID-19 pandemic. Extending from the Lombardo and Eichinger [12] concept, other researchers [20] have studied or applied learning agility in different contexts. For instance, De Meuse [20] studied the development and validation of the TALENTx7 Assessment, which is a psychological measure of learning agility [20]. This work expanded the original model into a 7-factor model, and later Burke and Mitchinson [21] developed a 9-factor model based on the original learning agility concept [21]. Norton [22] studied leadership flexibility and included learning agility as 1 of the definitional perspectives. Likewise, DeRue and Myers [23] built a framework for leadership development called PREPARE using learning agility as a key element. In Kaiser and Craig’s [24] view, learning agility is a meta-competency, meaning that it is the fundamental capacity that enables other technical competencies.

Other empirical studies show how learning agility can be applied. For example, Nesbit [25] used learning agility to assess leaders’ knowledge and skill acquisition and therefore assist with their behavioral repertoire expansion in self-directed leadership development. White and Shullman [26] discussed learning agility, especially the ability to accept the ambiguity of the working environment, as an indicator of effective leadership. When it comes to leadership across different managerial levels within an organization, learning agility was a positive predictor of leaders’ effectiveness in talent management [27]. De Meuse et al [27] noted that most people in leadership roles do not increase all-around competencies simultaneously when the job requirements change. Since it was reportedly rare in the management population to have high learning agility, it would be prudent for organizations to select individuals for key positions using the learning agility framework as a reference [27].

Studies on learning agility are often based on the premise that individuals actively seek professional development opportunities [13]. However, learning agility can also manifest within a specific circumstance where it is passively triggered (eg, the turbulent environment of the COVID-19 pandemic), which has not been previously discussed. In the case of COVID-19, both work content and format shifted. Individuals and organizations did not choose, but were forced, to develop and apply learning agility to survive. Learning agility can also exist on an organizational level. An organization’s reactions to turbulent environments and its strategies to solve novel challenges that impact many employees are also critical indicators of the long-term success of the organization [28]. Therefore, this study aims to describe the learning agility of L&D professionals in the life sciences sector when they encountered abrupt changes in their job requirements due to the emergence of the COVID-19 pandemic. Specifically, we answered the following research questions:

- How did L&D professionals in the life sciences sector respond to the changes in their work environment during the COVID-19 pandemic?
- How did L&D professionals in the life sciences sector demonstrate learning agility during remote working?

## Methods

### Study Design

This research study used a mixed methods approach to understanding how L&D professionals in the life sciences sector dealt with changes in their work due to the pandemic. Specifically, we were interested in their perceptions, solutions, and expectations for the future. Mixed methods research requires data triangulation from quantitative and qualitative data, which strengthens the construct validity of the study [29].

### Participants and Recruitment

Participants were recruited through an email list of Life Sciences Trainers & Educators Networks (LTEN) and its partner organizations, which included pharmaceutical companies, medical device manufacturing companies, biotechnology companies, and training and consulting companies with core services in the life sciences sector. The invitation emails were sent to the L&D departments of these organizations. Additional personnel who work closely with L&D departments, for example, the sales department, were also invited to participate. Salespersons were an important data source, as they are served by L&D departments and they directly interact with health care workers. After receiving the invitation emails, anyone who was interested in participating in this study could contact the researchers to complete informed consent, schedule an interview, or access the questionnaire through a link in the email. In the first phase of this study, we recruited 12 L&D professionals, whose experience ranged from 10 years to more than 30 years and held director or c-suite L&D positions in pharmaceutical, biotechnology, and medical device organizations, to participate in the interview. We intentionally focused our sampling for the interviews of experienced L&D practitioners, as they worked in the life sciences and health care L&D longer and witnessed the evolution of this area. Additionally, they had more connections with stakeholders, allowing them to have a macrolevel perspective. In the second phase of the study, we collected survey responses from 74 different individuals who held a variety of leadership positions.

### Qualitative Method

The interview was used to gather insights into overarching changes of professionals’ perceptions and mindsets about working remotely through the lens of learning agility. The semistructured interview was designed based on the existing literature and our subject matter experts’ understanding of the status quo of L&D in life sciences and health care. It contained 22 questions, with topics covering experiences and opinions, virtual solutions, digital literacy, and the future, making the conversations flow naturally. Interviewees’ responses and researcher’s notes served as data sources for the second phase of data collection. See Multimedia Appendix 1 for the interview questions.

### Quantitative Method

A follow-up survey questionnaire was designed based on the preliminary data collected through the interviews and expanded to 37 questions in total, with 8 (22%) demographic questions and 29 (78%) questions regarding 4 specific themes: organizational actions, remote working, L&D, and the future. Respondents were asked about their perceptions and expectations on these themes. See Multimedia Appendix 2 for the survey questions.

### Data Collection and Analysis

The interview data collection started during June 2020 and ended in August 2020. Interviews were conducted through videoconferencing or over the phone with 12 individuals that lasted approximately 30-60 minutes each. The survey data were collected from August to October 2020 using Qualtrics. Interviews were first transcribed and then coded and organized into groups of topics. These topics were expanded and specified into survey questions that were used in the second phase. The survey questions were designed for exploratory descriptive analysis with the intention to capture nuances of how professionals in a greater scale adapted their professional lives during the pandemic in contrast to exploring their psychological states or traits. Later, the survey responses and the interview data were coded and reorganized based on the learning agility framework adapted from Eichinger et al [30]. This framework of learning agility could be generalized as 4 key characteristics of learning agility: the willingness to adapt to new job requirements, the ability to handle jobs with increasing complexity, the ability to continuously learn new things, and the ability to overcome difficulties.

### Ethics Approval

In compliance with our university’s Institutional Review Board protocols (Study ID STUDY00009028), all participants signed an informed consent release prior to their data being collected. The research procedures were in accordance with the ethical standards of the responsible committee on human experimentation (institutional and national) and with the Declaration of Helsinki of 1975, as revised in 2000.

Participants were told that they did not have to answer any question they did not want to answer and could stop their participation at any time. All identities and data were kept confidential and anonymous.

## Results

### Demographics of Respondents

**Figure 1:**
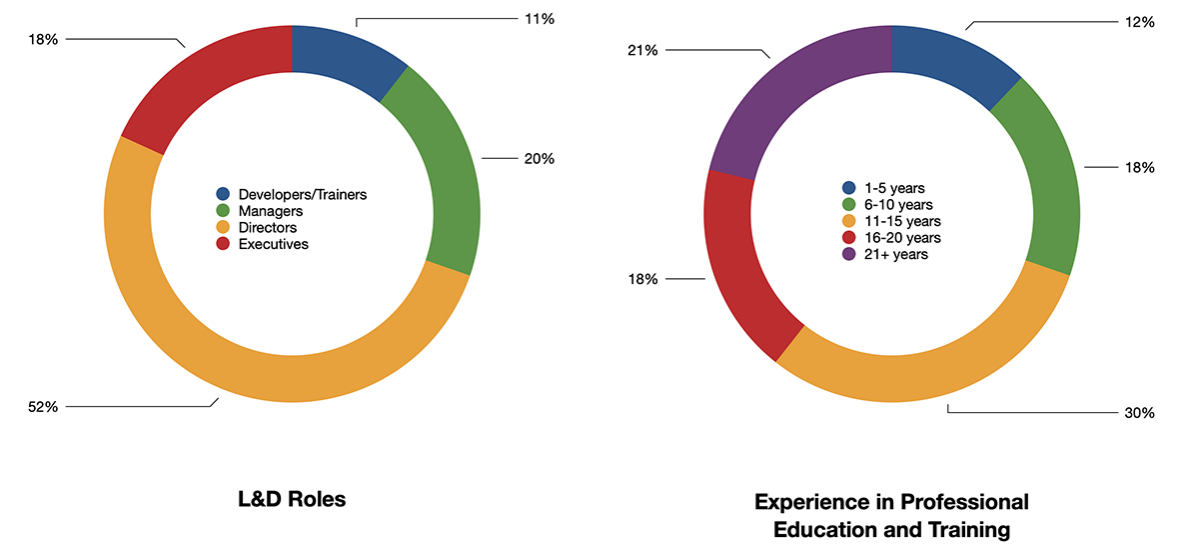
Information about respondents. L&D: learning and development.

The participants of this study were professionals (58 [88%] of 66) who held leadership roles in the L&D or equivalent departments of their life sciences organizations. As Figure 1 illustrates, 46 (70%) of 66 survey participants had 11+ years of experience, with 14 (30%) of these having 21 or more years of experience. More than 34 (51%) of the 66 participants were in director-level positions, 13 (20%) were managers, 12 (18%) were executives, and 7 (11%) were developers or trainers (Figure 1).

**Figure 2:**
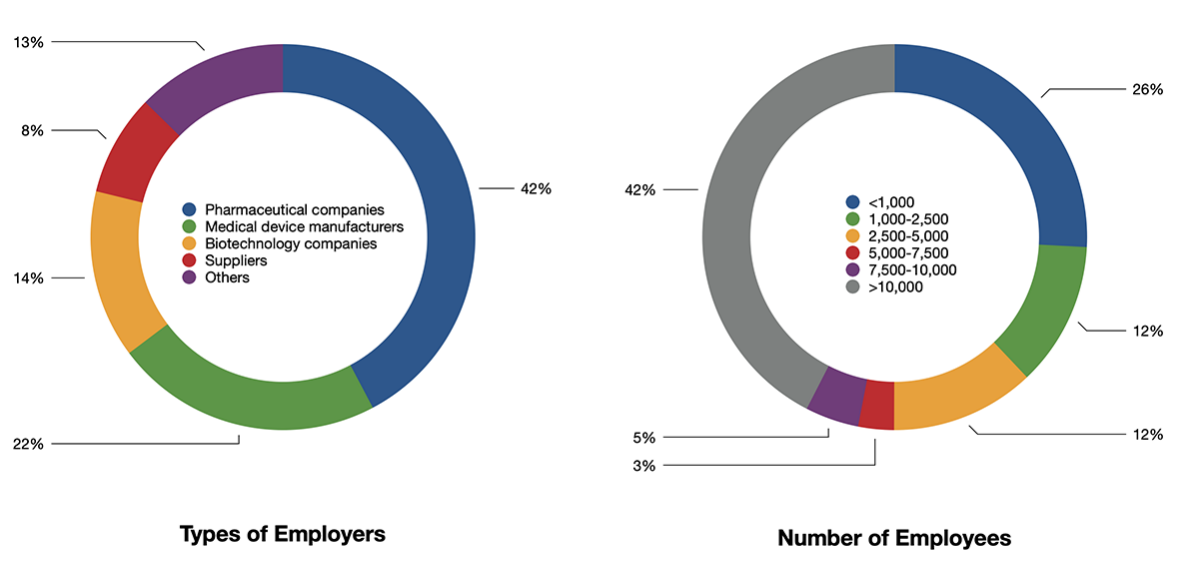
Information about respondents’ organizations.

Survey results also showed the respondents’ organization information. Respondents worked for medical device manufacturers (16 [23%] of 71 responses), pharmaceutical companies (30 [42%] of 71 responses), biotech companies (10 [14%] of 71 responses), suppliers (6 [9%] of 71 responses), and other types of organizations (eg, training companies, consulting firms, and labs). There were 28 (42%) of 66 respondents who worked in organizations that have more than 10,000 employees. Among the organizations of all respondents, 41 (62%) of 66 are entirely US based and 28 (42%) of 66 are directly involved in COVID-19 diagnostics or treatment (Figure 2). This information about participants’ leadership experience and type of organizations helped us interpret the survey and interview responses regarding their behaviors in response to the drastic shift of work, as well as their thoughts about the changes.

### How Organizations Responded to the Impact of COVID-19

The respondents were asked about their perceptions of their organizations during the work environment change: How quickly the organization leadership reacted to the pandemic, how they responded to emerging problems, and what they did to keep employees doing and feeling well.

#### Overall Responses

**Figure 3:**
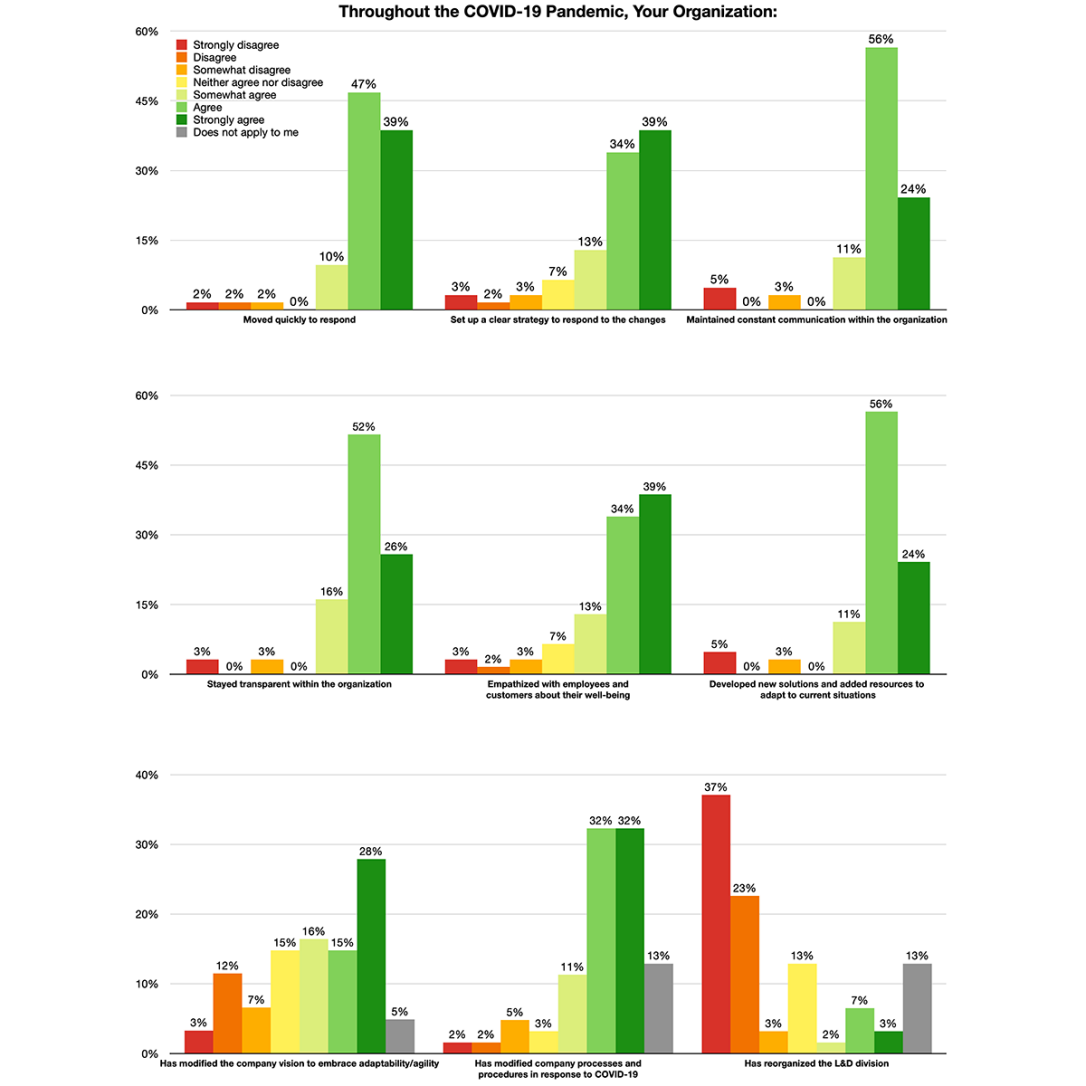
Levels of agreement to organizations’ responses to the pandemic. L&D: learning and development.

According to the majority of the respondents and interviewees (53-59 [86%-97%] of 62), they were appreciative of the overall response of the leadership during the pandemic (Figure 3). They agreed in varying degrees that the leadership moved quickly by setting up clear strategies, developing new solutions, modifying company processes and procedures, and adding resources to adapt to current situations. The leadership also remained in constant communication and were transparent within the organizations.

In other aspects, employees were not as satisfied. In terms of the modification of the company vision and removing barriers to embracing adaptability, these organizations were perceived as less successful by their employees. About 35-36 (58%-59%) of 61 respondents agreed with these items, but only 4 (7%) respondents strongly agreed that their organizations had removed barriers for them and customers. Moreover, fewer respondents (27-30 [44%-49%] of 61) agreed rather than disagreed or were indifferent that their organizations had maintained the pre-COVID-19 work atmosphere, increased decision-making speed, or modified the support system.

In the L&D department of these organizations specifically, 30-47 (56%-87%) of 54 respondents agreed that training- and curriculum-related work had been completely or partially moved online since beginning to work virtually. Such work tasks included, but were not limited to, onboarding processes, knowledge-based training, sales-related skills training, leadership skills training, soft/power skills training, and compliance training.

#### Responses to Employees’ and Customers’ Needs

**Figure 4:**
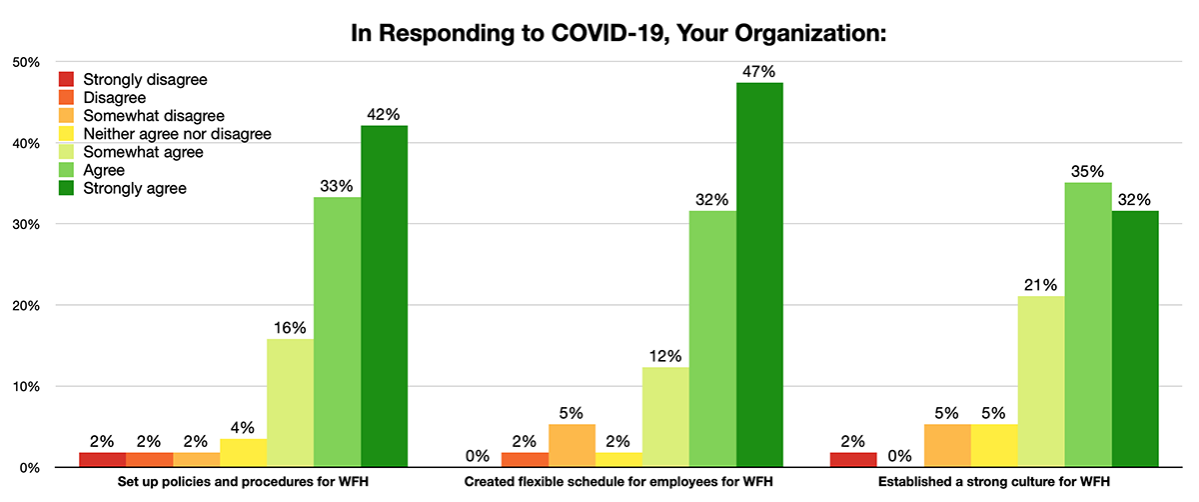
Levels of agreement to organizations’ support to remote working. WFH: working from home.

It was acknowledged by 47-55 (82%-96%) of 57 respondents that their organizations had created solutions to meet customers’ emerging needs by modifying or designing new products and services and leveraging technologies. Organizations had made efforts to allow employees to smoothly transition to the virtual working mode (Figure 4). According to the respondents’ ratings, 50-52 (88%-91%) of 57 of them agreed that their organizations had set up policies, created flexible schedules, and established strong cultures for the WFH situation.

Organizations also made efforts to indirectly meet customers’ needs by reskilling and upskilling their customer-facing and training-related employees. There were 46-47 (84%-86%) of 55 respondents who had participated in the reskilling and upskilling opportunities related to digital competencies either through virtual microlearning provided by their employers or by locating these upskilling resources on their own. The top 3 ranked uses of technologies perceived to be the most valuable were (1) tools for videoconferencing, (2) engaging customers, and (3) helping employees with information recall. Some interview participants reported that their organizations deployed classes for upskilling, such as virtual selling skills training and onboarding for just-in-time learning. However, a few others reported that they struggled as virtual sales and training limited customers’ and trainees’ engagement and the performance and training of soft skills for virtual sales were insufficient.

### How Individuals Responded to the Changes Caused by the Pandemic

With respect to the individual perspective of professionals in L&D, the respondents were asked about their perceptions of WFH experiences, customer-facing colleagues, and the changes in job responsibilities.

#### Respondents Treated Remote Working Positively

**Figure 5:**
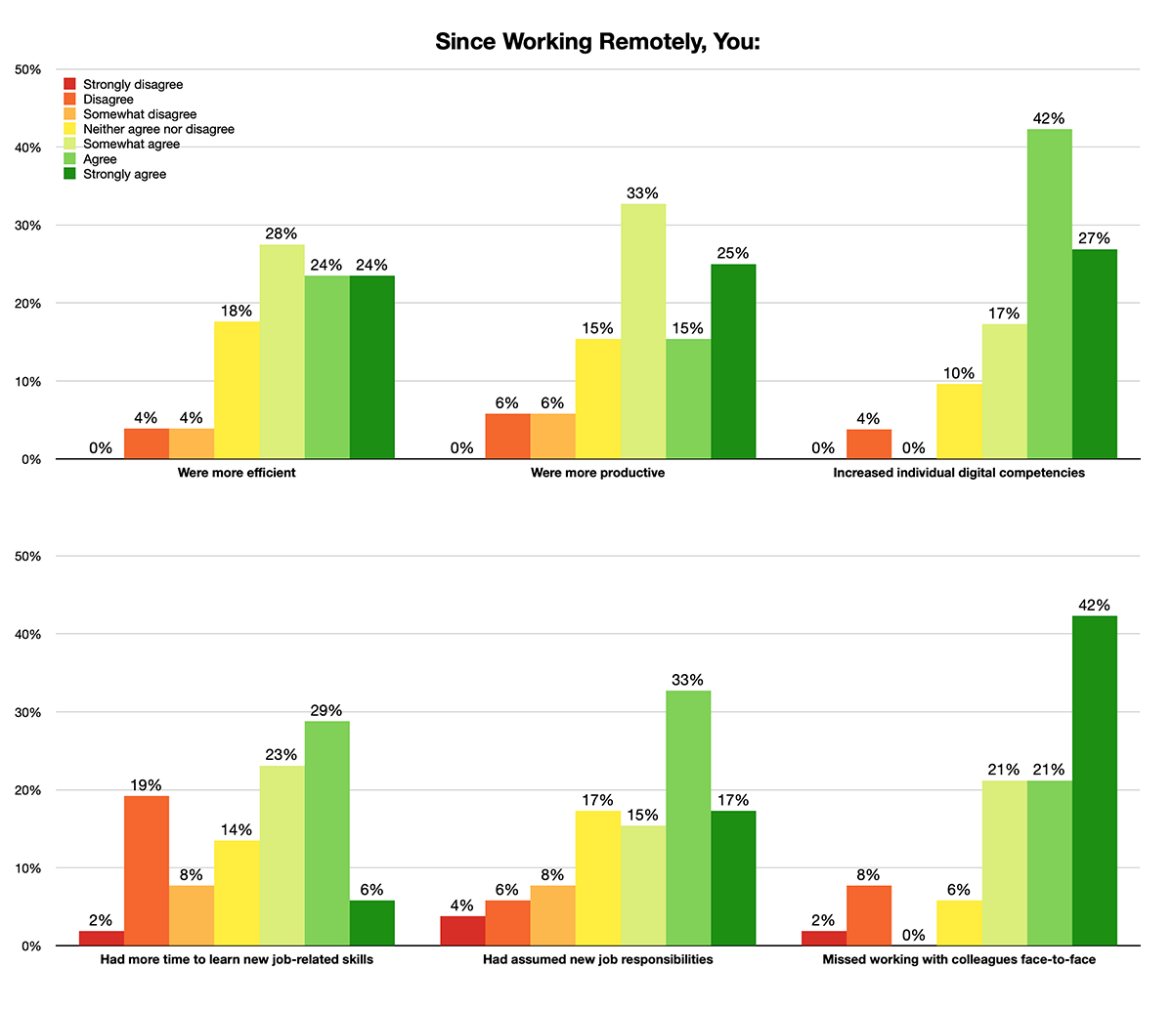
Levels of agreement to personal working experiences.

Although 31 (58%) of 53 respondents perceived WFH as temporary, 40 (70%) of 57 respondents still thought their organizations would consider WFH as a long-term strategy after the pandemic. They had gradually adapted to the new working mode and were mentally prepared for working remotely in the foreseeable future. Working remotely stimulated productivity and promoted the development of digital competencies (Figure 5). Approximately 38-45 (73%-87%) of 52 respondents reflected that they had become more efficient and productive and that they gained digital competencies since WFH began, yet 44 (85%) of them still missed working with colleagues face-to-face. Comparatively fewer respondents (30 [58%] of 52) perceived that they had more time to learn new things related to their jobs. A few participants addressed that they had been working remotely since before COVID-19, so they did not experience a significant change in their jobs.

#### The Change in Customer-Facing Employees’ Job Requirements

Among all the employees of these surveyed organizations, customer-facing employees experienced the most extreme changes in their day-to-day work. There were 45 (79%) of 57 respondents who perceived that customer-facing employees and field teams had more time to participate in trainings. More than 51 (90%) of the respondents perceived that these colleagues had increased digital selling competency. However, more respondents disagreed (26 [46%] of 57) than agreed (22 [39%] of 57) that digital sales allowed customer-facing employees to engage with more customers than traditional sales before COVID-19.

Moreover, 34 (65%) of 52 respondents realized they had assumed new and more job responsibilities. Several respondents stated in the comment area to explain their choices that demand services had far exceeded the ability to respond and that they could not keep up with the demand at the beginning. One respondent to a survey question (Multimedia Appendix 2, question 22) provided the following response that could explain why this discrepancy occurred:

The biggest gap is that clients thought virtual classes were easier to prepare, so the demand went up. However, the resources were limited…[and there was] high expectation from the clients [and their] insecure psychological state.

Direct quote from a participant

### Perceptions of “The New Normal”

**Figure 6:**
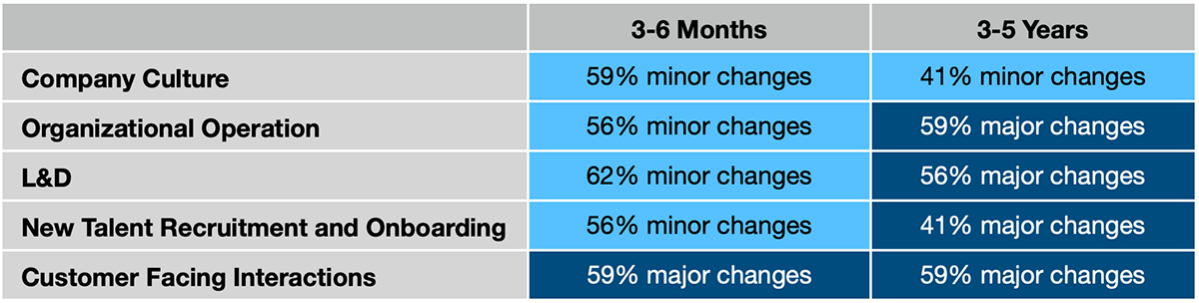
Prediction of the working environment in the short and the long term. L&D: learning and development.

Only 9 (17%) of 53 respondents believed that the field would go back to pre-COVID-19 training modes. The majority (31 [59%] of 53) believed that more organizational goals would be accomplished online even after COVID-19, and 9 (17%) of them believed that the pandemic triggered a paradigm shift and an evolution of this field. In the short-term (3-6 months), 30-33 (56%-62%) of 54 respondents thought that there would be minor changes in company culture, organizational operations, L&D, and recruitment and onboarding processes. There were 32 (59%) of these 54 respondents who thought that major changes would take place in customer-facing interactions. In other words, more respondents chose the minor-changes options than the major-changes options for the 4 aspects of the working environment, although in the long-term (3-5 years), more respondents (22-32 [41%-59%] of 54) tended to think there would be major changes versus minor changes in organizational operations, L&D, recruitment and onboarding, and customer-facing interactions (see Figure 6). It is worth noticing that although less than 27 (50%) of the respondents chose major changes for new talent recruitment and onboarding, it was still greater than the respondents who chose minor changes. See Multimedia Appendix 2 for the survey questions.

### How the Defining Characteristics of Learning Agility Were Represented in the Data

The leadership of these organizations reacted to the pandemic by shifting to remote working as soon as possible to maximize the security of their organizations, instead of resisting and waiting for the impact. This reflected their willingness to adapt to the new working conditions following the nationwide lockdown policies. One interviewee noted,

The people who provide education to the customers on-site, they immediately have switched to a virtual solution…We did everything we can…We figured out ways to do it right, [even though] it was not the most effective and efficient.

Direct quote from a participant

This response is aligned with the generally positive survey responses (see Table 1). On the individual level, participants mentioned that they initially experienced a phase of accepting the situation and then they endeavored to find out solutions. They also mentioned that a supportive team could make people resilient and proactive when faced with challenges.

As the job requirements changed, the work included new tasks and the task complexity increased. To handle jobs with increasing complexity, L&D and sales professionals were required to learn new things continuously, especially at the beginning stage of the transition. More than 65% (34/52) of the survey respondents agreed that they had assumed new job responsibilities since the onset of the pandemic. Organizations put extra emphasis on virtual trainings of various skills, sales, leadership, and soft skills. For L&D professionals, the amount of new training to be designed caused an increase in job responsibilities. Sales professionals also spent more time to participate in trainings, which left them with less time to work on their primary job tasks. For example, 1 (8%) of the 12 interviewees talked about the increasing frequency of their new employee orientations to address emerging issues in a timely manner, so they had to re-create orientation materials to suit shorter sessions. Training professionals reported that they learned new ways to achieve their goals of creating new orientation training. This, therefore, required more time to train professionals to design and deliver new content and for employees from other departments to help with the training. During this process, everyone explored new territories and learned something new, for example, virtual platforms and learning resources. Respondents reported that the most prominent gain in skills was digital competencies. Survey responses show that 45 (87%) of the 52 respondents agreed that they had increased individual digital competencies (Figure 5). In addition, almost every interviewee had referred to the adaptation to a new way of communication, collaboration, and operation through virtual platforms. There were difficulties many of them needed to overcome, since a significant number of senior employees were not familiar with virtual ways of working prior to the pandemic onset. In sum, the data revealed how the characteristics of learning agility were present in the work life of the professionals in our study in response to the pandemic. In Table 1, we identify 4 categories from the data that align with characteristics of learning agility: the willingness to adapt to new job requirements, the ability to handle jobs with increasing complexity, the ability to continuously learn new things, and the ability to overcome difficulties.

**Table 1:** Characteristics of learning agility and supporting evidence from the survey.^a^

Facet of learning agility	Supporting evidence in the data
Willingness to adapt to job requirements	Of 57 respondents, 52 (91%) agreed that their organizations set up policies and procedures for WFH^b^. Of 61 respondents, 27 (44%) agreed that their organizations had increased the speed of decision making. Of 61 respondents, 56 (91%) agreed that the leadership maintained constant communication within the organization. Of 61 respondents, 57 (93%) agreed that the leadership stayed transparent within the organization. Of 61 respondents, 52 (85%) agreed that the leadership empathized with employees and customers about their and their families’ well-being.
Ability to learn new things continuously	Of 62 respondents, 57 (92%) agreed that the leadership developed new solutions and added resources to adapt to current situations. Of 52 respondents, 45 (87%) agreed that they increased individual digital competencies since working remotely. Of 55 respondents, 46/47 (84%/85%) agreed that their organization provided reskilling/upskilling for the virtual training world. Of 56 respondents, 46 (82%) agreed that their organizations designed new products and services to meet clients’ current needs. Of 52 respondents, 30 (58%) agreed that they had more time to learn new things related to their jobs since working remotely. Of 61 respondents, 30 (49%) agreed that their organizations had modified the supporting system.
Ability to overcome difficulties	Of 61 respondents, 36 (59%) agreed that their organizations had modified the company vision to embrace adaptability/agility. Of 62 respondents, 47 (76%) agreed that their organizations had modified company processes and procedures in response to COVID-19. Of 57 respondents, 52 (91%) agreed that their organization created flexible schedule for employees to WFH. Of 56 respondents, 41 (73%) agreed that their organization had become more agile. Of 62 respondents, 36 (58%) agreed that their organizations had removed barriers. Of 62 respondents, 53 (85%) agreed that the leadership set up a clear strategy to respond to the changes.
Ability to handle jobs with increasing complexity	Of 56 respondents, 46 (82%) agreed that their organizations designed new products and services to meet clients’ current needs. Of 57 respondents, 45 (79%) agreed that their customer facing employees and field teams had more time to participate in training sessions. Of 51 respondents, 38 (75%) agreed that they were more efficient since working remotely. Of 52 respondents, 34 (65%) agreed that they had assumed new job responsibilities since working remotely. Of 57 respondents, 23 (40%) agreed that their customer facing employees and field teams had exhibited higher levels of productivity.

^a^All the items listed in the table are items whose “agrees” options were selected by more respondents than “disagrees” options, even though some “agrees” responses were lower than 50%. See Multimedia Appendix 2 for survey questions.

^b^WFH: working from home.

## Discussion

### Principal Results

The interview and survey results indicated that L&D professionals were overall positive in their perceptions of their organization’s leadership, their colleagues, and themselves in terms of finding solutions and supporting one another. The majority of respondents reported increased productivity and opportunities to reskill and upskill during WFH. In addition, they upgraded their digital competencies, especially technologies for videoconferencing, engaging customers, and helping learners’ information recall. In addition, this study revealed some insights that the framework adapted from Eichinger et al [30] did not discuss. Their framework was developed to evaluate an employee’s leadership potential and effectiveness. Our findings suggest that elements of learning agility were demonstrated by most survey participants in response to a highly disruptive crisis, when the working mode abruptly shifted. This study provides insights into how the pandemic created a context that sparked a different entry point (ie, the drive for job survival) into the learning agility development cycle. Results also provide insights into how the COVID-19 crisis demanded professionals’ learning agility to both self-initiate their own learning and to support the learning agility of others in the organization.

### Limitations and Future Directions

In the quantitative component of the study, 74 participants responded to the survey, which represents ~5% of the sample pool of 1500 people. It is below the average of medium-length web-based surveys (12-25 questions)—less than 10% [31-33]. Given that our survey consisted of 37 questions, it was reasonable to have a lower response rate, as the length of a survey has a negative influence on the response rate [33,34]. One possible direction for future research is to increase the sample size of participants by expanding the survey to a larger membership body [35]. In addition, the participating population of this study was skewed to high management roles and training professionals. Sales professionals, however, are the trainees closely interacting with these training professionals and are as much impacted by the pandemic as training professionals. Therefore, future studies could shift the recruiting focus to first-line sales professionals to investigate how specifically the overall life cycle of training and education for sale professionals changed.

Another possible direction for future studies is to dig deeper into the learning agility framework by addressing the mental and emotional aspects. To implement this idea, we need to ask more why and how questions about people’s motivations and ways of predicting and solving problems, and to intentionally differentiate people’s pandemic leadership behaviors from those of the prepandemic period.

Last but not least, the transition to the new way of working that we have observed was reactionary at the core. It would be worthwhile to see whether the L&D professionals would adopt a more proactive approach to learning agility as the pandemic subsides or whether they drift back to the old normal.

### Conclusion

Prior research has explored current and future trends in life sciences training [36], noting trends for more remote L&D initiatives. This study examined a more specific circumstance where life sciences L&D professionals faced unprecedented challenges, requiring a sudden and unexpected shift to remote work. This study explored the organizational and individual reactions of life sciences organizations toward the pandemic and the shift in the working environment that it entailed. In retrospect, the first 3 months of the pandemic were a survival phase for most of the organizations in the life sciences sector. The leadership lacked experience and confidence in their solutions during such a turbulent time. The next several months presented an adoption phase, which was marked by a collective will to make change. This phase aligns with the period during which we collected our data and, as such, reflects how learning agility appeared at the organizational and individual levels during this time. Looking toward the future as vaccines are more prevalent, organizations are now more competent to shift into a proactive phase. They have more experience dealing with unpredictable and abrupt changes in the global environment. The changes in work life, due to the COVID-19 crisis, may have pushed the development of the industry 5-7 years forward into the future in a few months [37]. The forces that prompted the development include the organization’s drives for financial success, customer demands, and, more prominently, the practitioners’ adaptation and agility. They demonstrated a willingness to be agile that may not have emerged as quickly outside of the pandemic crisis. Future work should investigate how life sciences L&D organizations structure their remote work and remote training in a post-COVID-19 world. Both our interview and survey results predicted that a mix of live and virtual solutions for working, training, and learning would emerge to better suit the ever-changing market in life sciences organizations.

## Appendix

Multimedia Appendix 1 Interview questions.

Multimedia Appendix 2 Survey questions.

## Abbreviations

L&D: learning and development
LTEN: Life Sciences Trainers & Educators Networks
WFH: working from home
