# Supplementary material for: Learning Agility of Learning and Development Professionals in the Life Sciences Field During the COVID-19 Pandemic: Empirical Study
Source: Interact J Med Res. 2022 Apr 26;11(1):e33360. doi: 10.2196/33360 (PMC9045484; doi:10.2196/33360)
Supplement: Multimedia Appendix 1 [file ijmr_v11i1e33360_app1.docx]

**Multimedia Appendix 1. Interview Questions**

**[Background info]**

1. Please start by telling me about yourself, your position, and your company.
2. How long have you been involved with life sciences training?

**[Experiences and Opinion info]**

1. Please describe what your training department experienced as you moved into the COVID-19 pandemic.
2. What did your sales department (field and in-house sales team) experience?
3. Can you list some tools that were especially valuable to your learning and/or sales department during this pandemic? Were there other tools that were no longer as valuable?
4. Were there any particular training skills that became more valuable as a result of the pandemic?
5. How do you think your training roles and portfolios might change in the future, to create the agility that may have been missing at this moment?
6. What is your biggest take away from this experience from both training and sales perspectives?
7. Based on recent experiences, is there anything specifically you are doing to up-skill yourself and/or your team?

**[Virtual solution-related Items]**

1. As a result of this experience, do you think virtual solutions, such as video conferencing, online courses, and teleconferencing, and perhaps others will become more valuable? Did you use any of these during this time? What about your field sales team? How do you think virtual solutions have helped them reach out to customers virtually?
2. Do you believe that technologically supported remote learning can be as effective as face-to-face options? Are there features or functionality that are missing? What about your field sales team? What do you think their thoughts on this would be?
3. Do you need to assess learning performance differently when learners and educators connect virtually? What differences in assessment might you recommend?
4. How have learners responded differently to technologically supported remote learning during this pandemic? Will these changes be sustained after the pandemic is controlled?
5. If you could transition to high-quality virtual solutions, what benefits might be realized by your learners, your staff, and your organization?

**[Thoughts on digital literacy]**

In this next set of questions, we’ll be talking about “digital literacy,” which we will define as finding and consuming digital content, creating and communicating or sharing it.

1. Do you think your learners (employees and customers) are lacking digital literacy skills? If so, can you share some examples?
2. What kind of digital literacy do you want your audiences (internal and customer) to be applying?
3. If cost and other resources were not factors, what tools, approaches, or technologies would you like to adopt to improve digital literacy, and why?

**[Thoughts on the future]**

1. Do you think the field will ever go back again to the pre-COVID-19 training mode?
2. Do you plan to embrace the paradigm shift to learning in the new normal, or will you return to familiar practices?
3. What do you think will happen in the next 3 months in the learning industry, your organization, and your L&D department?
4. Where do you see the life science training field going in the next year? Five years?

**[Wrap up]**

1. Anything else you would like to share?
