# Supplementary material for: Learning Agility of Learning and Development Professionals in the Life Sciences Field During the COVID-19 Pandemic: Empirical Study
Source: Interact J Med Res. 2022 Apr 26;11(1):e33360. doi: 10.2196/33360 (PMC9045484; doi:10.2196/33360)
Supplement: Multimedia Appendix 2 [file ijmr_v11i1e33360_app2.docx]

**Multimedia Appendix 2. Survey Questions**

Your participation is voluntary, and you may decide to stop at any time.  You do not have to answer any questions that you do not want to answer.
 
Are you willing to participate in this study?

- Yes, I consent to participate in the study
- No, I do not consent to participate in the study

Skip To: End of Survey If Consent for Exempt Research The Pennsylvania State University Title of Project: Learning Agility:... = No, I do not consent to participate in the study

**Demographic**

Q1 What type of company best describes your current employer?

- Medical device manufacturer
- Pharmaceutical company
- Bio-tech company
- Suppliers
- Other, please indicate ________________________________________________

Skip To: End of Survey If What type of company best describes your current employer? = Suppliers

Q2 Which best describes your L&D role in the organization?

- Developer/ Trainer
- Manager
- Director
- Executive

Q3 How many employees does your organization have?

- Less than 1,000
- 1,000 to 2,500
- 2,500 to 5,000
- 5,000 to 7,500
- 7,500 to 10,000
- More than 10,000

Q4 Who is the audience for your work?

- Internal employees
- External customers
- Both

Q5 What demographics are you responsible for?

- An entire US Organization
- An entire Global Organization
- Other ________________________________________________

Q6 Do you have non-commercial responsibility?

- Yes
- No
- Other ________________________________________________

Q7 Is your organization directly involved with COVID-19 diagnostics, treatment and/or other related solutions?

- Yes
- No

Q8 How long have you been involved in professional education and training?

- 1-5 years
- 6-10 years
- 11-15 years
- 16-20 years
- 21 years or more

**Organization**

Q9 Throughout the COVID-19 pandemic, the leadership in your organization:

|  | Strongly disagree | Disagree | Somewhat disagree | Neither agree nor disagree | Somewhat agree | Agree | Strongly agree |
| --- | --- | --- | --- | --- | --- | --- | --- |
| Moved quickly to respond |  |  |  |  |  |  |  |
| Set up a clear strategy to respond to the changes |  |  |  |  |  |  |  |
| Maintained constant communication within the organization |  |  |  |  |  |  |  |
| Stayed transparent within the organization |  |  |  |  |  |  |  |
| Empathized with employees and customers about their wellbeing and the wellbeing of family members |  |  |  |  |  |  |  |
| Developed new solutions and added resources to adapt to current situations |  |  |  |  |  |  |  |

Q10 Please feel free to add comments to clarify any of your responses above. (Optional):

________________________________________________________________

Q11 Throughout the COVID-19 pandemic, your organization:

|  | Strongly disagree | Disagree | Somewhat disagree | Neither agree nor disagree | Somewhat agree | Agree | Strongly agree |
| --- | --- | --- | --- | --- | --- | --- | --- |
| Has modified the company vision to embrace adaptability/agility |  |  |  |  |  |  |  |
| Has maintained the pre COVID-19 work atmosphere |  |  |  |  |  |  |  |
| Has modified company structure in response to COVID-19 |  |  |  |  |  |  |  |
| Has modified the structure of the L&D division |  |  |  |  |  |  |  |
| Has increased the speed of decision making |  |  |  |  |  |  |  |
| Has removed barriers |  |  |  |  |  |  |  |
| Has an increased sense of social responsibility |  |  |  |  |  |  |  |
| Has modified the supporting system |  |  |  |  |  |  |  |
| Has more people working remotely |  |  |  |  |  |  |  |

Q12 Please feel free to add comments to clarify any of your responses above. (Optional):

________________________________________________________________

________________________________________________________________

Q13 Looking back on all that has happened since the onset of COVID-19, what are the most important lessons for professionals in our field?

________________________________________________________________

**Working Remotely**

Q14 In responding to COVID-19, your organization:

|  | Strongly disagree | Disagree | Somewhat disagree | Neither agree nor disagree | Somewhat agree | Agree | Strongly agree |
| --- | --- | --- | --- | --- | --- | --- | --- |
| Set up policies and procedures for working from home |  |  |  |  |  |  |  |
| Created flexible schedules for employees to work from home |  |  |  |  |  |  |  |
| Established a strong culture for working from home |  |  |  |  |  |  |  |
| Views working from home as temporary |  |  |  |  |  |  |  |
| Will continue to embrace working from home even after COVID-19 |  |  |  |  |  |  |  |
| Has become more agile |  |  |  |  |  |  |  |

Q15 Please feel free to add comments to clarify any of your responses above. (Optional):

________________________________________________________________

Q16 Since COVID-19, your organization has:

|  | Strongly disagree | Disagree | Somewhat disagree | Neither agree nor disagree | Somewhat agree | Agree | Strongly agree | Does not apply to me |
| --- | --- | --- | --- | --- | --- | --- | --- | --- |
| Modified products and services to meet clients' current needs |  |  |  |  |  |  |  |  |
| Designed new products and services to meet clients' current needs |  |  |  |  |  |  |  |  |
| Leveraged technologies to meet clients' current needs |  |  |  |  |  |  |  |  |

Q17 Please feel free to add comments to clarify any of your responses above. (Optional):

________________________________________________________________

Q18 Since COVID-19, your sale representatives:

|  | Strongly disagree | Agree | Somewhat agree | Neither agree nor disagree | Somewhat agree | agree | Strongly agree | Does not apply to me |
| --- | --- | --- | --- | --- | --- | --- | --- | --- |
| Have increased Digital Selling competency |  |  |  |  |  |  |  |  |
| Have engaged with more clients than before |  |  |  |  |  |  |  |  |
| Have exhibited higher levels of productivity |  |  |  |  |  |  |  |  |
| Have more time to participate in training sessions |  |  |  |  |  |  |  |  |

Q19 Please feel free to add comments to clarify any of your responses above. (Optional):

________________________________________________________________

Q20 Are you personally working remotely as a result of COVID-19?

- Yes
- No

Display This Question:

If Are you personally working remotely as a result of COVID-19? = Yes

Q21 Since working remotely, you:

|  | Strongly disagree | Disagree | Somewhat disagree | Neither agree nor disagree | Somewhat agree | Agree | Strongly agree |
| --- | --- | --- | --- | --- | --- | --- | --- |
| Are more efficient |  |  |  |  |  |  |  |
| Are more productive |  |  |  |  |  |  |  |
| Have more time to learn new things related to your job |  |  |  |  |  |  |  |
| Have assumed new job responsibilities |  |  |  |  |  |  |  |
| Have more respect for your colleagues |  |  |  |  |  |  |  |
| Miss working with your colleagues face-to-face |  |  |  |  |  |  |  |
| Increased individual digital competencies |  |  |  |  |  |  |  |

Display This Question:

If Are you personally working remotely as a result of COVID-19? = Yes

Q22 Please feel free to add comments to clarify any of your responses above. (Optional):

________________________________________________________________

**Learning and Development**

Q23 Since COVID-19, your organization has:

|  | Strongly disagree | Disagree | Somewhat disagree | Neither agree nor disagree | Somewhat agree | Agree | Strongly agree | Does not apply to me |
| --- | --- | --- | --- | --- | --- | --- | --- | --- |
| Hired people with new talents in L&D to meet clients' current needs |  |  |  |  |  |  |  |  |
| Has been able to generate commitment to the company in new hires, despite changes in the onboarding process |  |  |  |  |  |  |  |  |
| Provided upskilling for the virtual training world |  |  |  |  |  |  |  |  |
| Provided reskilling for the virtual training world |  |  |  |  |  |  |  |  |

Q24 Please feel free to add comments to clarify any of your responses above. (Optional):

________________________________________________________________

Q25 Has your L&D unit been downsized?

- Yes
- No

Display This Question:

If Has your L&D unit been downsized? = Yes

Q26 What factors contributed to the downsizing? Select all that apply.

- Budget cut
- Need people with competencies that current employees did not possess
- Need people with visions or philosophies that current employees did not possess
- Other ________________________________________________

Q27 Has your organization, outside of L&D, been downsized?

- Yes
- No

Display This Question:

If Has your organization, outside of L&D, been downsized? = Yes

Q28 What factors contributed to the downsizing outside of your L&D? Select all that apply.

- Budget cut
- Need people with competencies that current employees did not possess
- Need people with visions or philosophies that current employees did not possess
- Other ________________________________________________

Q29 How has COVID-19 impacted your organization's L&D budget during 2020?

- Increased significantly
- Increased somewhat
- No change
- Decreased somewhat
- Decreased significantly

Q30 How has COVID-19 impacted your organization's L&D downsizing?

- Cost financial deficient
- Require a new set of competencies that current employees do not process
- Both
- We haven't downsized, and will not downsize
- We haven't downsized, but it will happen in the near future
- Other ________________________________________________

Q32 How has COVID-19 impacted your organization's learning and development?

|  | Has not changed | Nothing has changed because it was all online prior to COVID | We moved some components online | We moved everything online | Postponed until we can do this live | Does not apply to me |
| --- | --- | --- | --- | --- | --- | --- |
| Onboarding process |  |  |  |  |  |  |
| Knowledge-based training |  |  |  |  |  |  |
| Sales related skills training |  |  |  |  |  |  |
| Leadership related skills training |  |  |  |  |  |  |
| Soft/ Power skills training |  |  |  |  |  |  |
| Compliance training |  |  |  |  |  |  |

Q33 What are the most valuable technologies in terms of training your employees and customers online throughout the pandemic? Please drag no more than 5 items in the left-hand column to the ranking box placing with the most important items at the top. The box will expand on its own.

| Ranking |
| --- |
| ______ Video Conferencing Tool, such as Zoom and MS Teams |
| ______ Virtual Collaboration Space, such as Conceptboards |
| ______ Virtual Social Spaces, such as LinkedIn |
| ______ Applications that increase learner engagement, such as Viva Engage |
| ______ Applications that help learner with information recall, such as Kahoot, QStream, and Umu |
| ______ LMS or CMS |
| ______ Emerging technologies, such as VR and AR |
| ______ Platform that leverages AI |
| ______ Video production tools |

Q34 What is your overall impression of your company's response to COVID-19 in L&D and beyond?

________________________________________________________________

**Future**

Q35 Do you believe our field will ever go back to pre-COVID training mode?

- Yes.
- No, while some things will stay the same, more of our goals will be accomplished online.
- No, I believe this triggers a paradigm shift and our field will evolve rapidly.
- No... (Please provide an alternative answer in the text box below.)

________________________________________________

- I am not sure.

Q36 What do you perceive will happen in the next 3-6 months?

|  | Nothing will change | Minor changes | Major changes | I am not sure |
| --- | --- | --- | --- | --- |
| Company Culture |  |  |  |  |
| Operations |  |  |  |  |
| Learning & Development |  |  |  |  |
| New talent recruitment & onboarding |  |  |  |  |
| Sales |  |  |  |  |

Q37 What do you perceive will happen in the next 3-5 years?

|  | Nothing will change | Minor changes | Major changes | I am not sure |
| --- | --- | --- | --- | --- |
| Company Culture |  |  |  |  |
| Operations |  |  |  |  |
| Learning & Development |  |  |  |  |
| New talent recruitment & onboarding |  |  |  |  |
| Sales |  |  |  |  |

Q38 What makes you most proud of your company's reaction to COVID-19 L&D and beyond?

________________________________________________________________

Q39 What has been your organization biggest mistake in response to COVID-19 L&D and beyond?

________________________________________________________________
